# RWD insights into disease progression

**DOI:** 10.1002/alz.084542

**Published:** 2025-01-09

**Authors:** Maria Eriksdotter

**Affiliations:** ^1^ Karolinska Institutet, Stockholm Sweden

## Abstract

**Background:**

The new anti‐Aβ antibody drugs aducanumab and lecanemab (approved in the US, not yet in Europe) must be followed up closely and regularly long‐term. Previous knowledge on progression of AD in routine clinical settings longterm is crucial when introducing new dementia medications. The Swedish national quality database on dementia/cognitive disorders, SveDem, where data on different dementia disorders at the time of the dementia diagnosis since 2007 and on mild cognitive impairment (MCI) since 2021 with annual follow‐ups of MMSE scores can provide this unique information.

**Methods:**

SveDem, www.svedem.se is to date the largest database in the world on different dementia disorders and from 2021 also on MCI. The registry includes individuals at the time of the MCI or dementia diagnosis with annual follow‐ups, at present with >122, 000 persons, who are followed through the chain of care. By linking SveDem to other registers, data on diagnostics including biomarkers, comorbidities, treatment, care, mortality and prognosis can be obtained.

**Results:**

From SveDem have shown that mortality in dementia disorders over a 10‐year time period is decreasing, partly associated with changes in drug prescribing practices. Among the different dementia disorders, SveDem data show that AD has the best survival while the highest mortality is found in persons with DLB/PDD. Follow‐up data on use of the current dementia drugs choline esterase inhibitors (ChEIs) in an AD population of > 17 000 persons showed ChEIs to be associated with small but persistent cognitive benefits long term (5 years). Moreover, based on SveDemdata, ChEI treatment was found to be associated in an AD population with reduced risk for myocardial function, stroke, heart failure, kidney dysfunction and overall mortality. Additional data on disease progression will be presented.

**Conclusion:**

Clinical routine long‐term data on large populations of persons with AD (and other dementia disorders) treated with or without current dementia drugs, on prognosis, progression and mortality already exist to be used for long‐term comparisons with new approved AD medications. Moreover, the quality data base SveDem collects data regularly and will start gathering data on the new anti‐amyloid dementia drugs on effects/progression if approved in Europe.

